# Association of electrocardiogram alterations of rescuers and performance during a simulated cardiac arrest: A prospective simulation study

**DOI:** 10.1371/journal.pone.0198661

**Published:** 2018-06-14

**Authors:** Lucas Tramèr, Christoph Becker, Seraina Hochstrasser, Stephan Marsch, Sabina Hunziker

**Affiliations:** 1 Medical Intensive Care Unit, University Hospital Basel, Basel, Switzerland; 2 Medical Communication and Psychosomatic Medicine, University Hospital Basel, Basel, Switzerland; 3 Department of Emergency Medicine, University Hospital Basel, Basel, Switzerland; Azienda Ospedaliero Universitaria Careggi, ITALY

## Abstract

**Background:**

Performance of cardiopulmonary resuscitation (CPR) causes significant mental stress for rescuers, especially if performed by inexperienced individuals. Our aim was to study electrocardiogram (ECG) alterations in rescuers and its association with gender and CPR performance.

**Methods:**

We included 126 medical students in this prospective, observational simulator study. Each student was equipped with a 3-lead continuous ECG device tracking the individual electrocardiographic output before, during and after CPR. We analyzed variations in heart rate, heart-rate variability (HRV) and ST- and T-wave morphology.

**Results:**

Compared to baseline, mean heart rate (bpm) significantly increased during resuscitation and again decreased after resuscitation (from 87 to 97 to 80, p<0.001). Heart-rate variability (the standard deviation of all N-N intervals, SDNN) (ms^2^) showed the opposite pattern, decreasing during resuscitation and increasing after resuscitation (117 to 92 to 93ms, p<0.001). Abnormalities in T-waves and ST-segments were observed in 29.4% of participants. Maximal heart rate (r = 0.25, p = 0.046) as well as heart rate reactivity (r = 0.7, p<0.001) correlated with hands-on time, a measure of CPR performance. Compared to males, female rescuers had a significantly higher maximal heart rate (136bpm vs. 126bpm, p = 0.008) and lower HRV (SDNN 102 vs. 119ms, p = 0.004) and tended to show more abnormalities in T-waves and ST-segments (36% vs. 21%, p = 0.080).

**Conclusion:**

CPR causes significant ECG alterations in healthy medical students with ST-segment and T-wave abnormalities, with more pronounced effects in females. Clinical implications of these findings need to be further investigated.

## Introduction

A cardiac arrest is an emergency situation that causes mental stress to all involved rescuers. The extent of stress-response to an acute stressor and the individual appraisal of an emergency situation shows a high inter-individual variability and is influenced by genetic factors, age, environment and gender [1]. Physical and mental stress lead to the activation of the hypothalamic–pituitary–adrenal axis (HPA) with consequent cortisol secretion from the adrenal glands also known as “fight or flight” reaction. Moreover, stress activates the sympathetic nervous system. These factors lead to a physiological response to acute mental stress in healthy subjects, which includes an increase in cardiac output, heart rate and systemic vascular resistance and thus an increase of cardiac afterload [2, 3]. The largest stress responses seem to be triggered by both uncontrollable and social-evaluative performance tasks [4].

Cardiopulmonary resuscitation (CPR) is a clinical example of such an uncontrollable task. A simulated cardiac arrest might cause significant mental stress, which can adversely impact resuscitation performance [5]. Effective teamwork and leadership improves CPR performance [6–8]. In the past, mental stress during CPR has been analyzed using subjective stress markers and physiological parameters such as heart rate, heart rate variability and cortisol levels [9]. Interestingly, in these studies, gender differences were observed with higher subjective stress levels experienced by female students [5]. However, while several studies investigated the effect of stress measured by vital signs and blood hormone levels on CPR performance little is known about changes in heart physiology in response to CPR-associated stress.

Herein, we aimed to further evaluate the impact of a simulated cardiac arrest on ECG data in healthy rescuers. Particularly, we were interested in studying potential stress induced ECG variations such as ST segment or T wave alterations [2]. As CPR may also cause physical stress, we expected to see ECG dynamics reflecting both mental and physical stress of the participants. In addition, we investigated associations between ECG alterations and leadership role, gender and CPR performance. Our hypothesis was that a simulated cardiac arrest would cause significant alterations in parameters of autonomic cardiac regulation and show signs of acute myocardial stress during resuscitation.

## Methods

### Study design

This prospective, observational study was performed between December 2011 and April 2012 at the Simulator Center of the University Hospital in Basel, Switzerland. We recruited medical students of the University of Zurich, offering the possibility to test their know-how in emergency medicine in a realistic CPR simulation. Participants were blinded to the context of the scenario and the hypotheses of the study.

The study was approved by the local institutional review board (Ethikkommission Nordwestschweiz, EKNZ), and all participants gave written informed consent.

### Simulator and resuscitation scenario

For this study, we used a high-fidelity mannequin with the possibility of remote control of vital signs and allowing realistic interactions (Human Patient Simulator, METI, Sarasota, FL, USA).

Our study consisted of two consecutive exercises in which the participants faced a simulated cardiac arrest. A randomly selected participant was assigned the role of group leader. The remaining students were asked to follow the leader’s instructions during the whole simulation. The mannequin simulated a cardiac arrest and the students performed CPR and manual external defibrillation. The cardiac arrest scenario lasted for at least 3 minutes independent of the actions taken by the team. In every group the simulated patient survived the cardiac arrest.

### Assessment of electrophysiological parameters

All participants were equipped with a continuous 3-lead ECG recording during the whole simulation. Electrophysiological parameters were assessed at different time-points during the simulation, including immediately before, during and after CPR.

We assessed heart rate (HR) and heart rate variability (HRV) which are considered direct markers of supraventricular sympathetic drive[10]. During mental stress, HR increases and total HRV decreases as a consequence of increased sympathetic drive[1]. HRV was measured using the standard deviation of all normal to normal intervals (SDNN) (an estimate of overall HRV) and the square root of the mean squared differences of successive normal to normal intervals (RMSSD) (an estimate of short-term HRV) [11].

Additionally, we assessed the continuous 3-lead ECG recordings for acute variations in ST-segment and T-wave morphology. T-wave amplitude (TWA) is an accurate measure for ventricular sympathetic activity and decreases consistently in response to physical and mental stress[10]. ST segment depressions, a correlate of myocardial ischemia, are usually seen in patients with coronary artery disease. However, ST-segment variations can also be seen in healthy subjects under mental stress, possibly mediated by vasospasms of the coronary arteries[2].

### Outcomes

The primary endpoints of this study were dynamic ST-segment and T-wave alterations. Secondary endpoints included variations in HRV (SDNN, RMSSD) and heart rate (minimal, maximal, mean and reactivity). Heart rate reactivity was defined as the difference between maximal and minimal HR. We compared these variables before, during and after CPR on an individual level and correlated the changes with performance of CPR. In addition, we analyzed the effect of gender, designated leadership and chest compressions on these parameters. CPR performance was assessed using hands-on time, defined as the duration of uninterrupted chest compressions or defibrillation of shockable rhythms in the first 180 seconds after the onset of the cardiac arrest. The first meaningful measure (FMM) was defined as the time elapsed until CPR was started, after the onset of cardiac arrest (either defibrillation, chest compression or ventilation).

### Data analysis

We divided the simulation into different time-sequences including 10 minutes before the simulated cardiac arrest (“before resuscitation”), the duration of cardiopulmonary resuscitation (“during resuscitation”), and 30 minutes after “awakening”of the mannequin (“after resuscitation”).

The analysis of the electrocardiographic recordings was performed using *Philips Zymed Holter Serie 1810 2*.*9*.*2*. CPR-related actions (hands-on time) were coded second by second using video recordings of the simulations.

### Statistical analysis

We investigated dynamics of different ECG parameters before, during and after resuscitation using an analysis of variance (ANOVA). Individual differences across time-periods and subgroups were compared using paired t-tests.

To assess the association of the different stress markers with CPR performance, we performed linear regression analyses. For these analyses, individual stress data was analyzed in relation to group performance with hands-on time as a linear group-level outcome. All analyses were performed using STATA 12.1 (Stata Corp, College Station, TX). P values < 0.050 were considered to indicate statistical significance.

## Results

### Study population and CPR performance

A total of 126 students (56 male, 70 female) volunteered for participation in this study (Table 1). The first meaningful measure (FMM) was at a mean of 56 s (±19). All teams undertook the first meaningful measure in under 100 s. Two groups started chest compressions later than 180 s after the onset of cardiac arrest. The mean hands-on time of all groups within the first 180 s was 101 s (±28).

**Table 1:** Baseline characteristics of resuscitation teams.

**Participants**	
Total (n)	126
Female (n)	70
Male (n)	56
**CPR team performance**	**sec**
First meaningful measure, mean (SD)	56 (19)
Hands on time at 60 sec, mean (SD)	8 (10)
Hands on time at 120 sec, mean (SD)	54 (20)
Hands on time at 180 sec, mean (SD)	101 (28)
Hands on time at 240 sec, mean (SD)	147 (34)
Hands on time at 300 sec, mean (SD)	197 (37)
Time to start CPR, mean (SD)	68 (44)
Time to defibrillation, mean (SD)	183 (73)
Time to ventilation, mean (SD)	77 (26)
Time to adrenaline, mean (SD)	265 (60)

CPR: Cardiopulmonary resuscitation

### Heart rate and heart rate variability

First, we measured HR and HRV in all individuals at three different time points, namely before, during and after resuscitation (Table 2). Mean heart rate increased significantly from 87 bpm before resuscitation to 97 bpm during resuscitation and again decreased to 80 bpm after resuscitation (p<0.001). SDNN decreased from 117 ms to 92 ms during CPR and increased to 93 ms after CPR (p<0.001). RMSSD showed the same pattern as SDNN without reaching statistical significance (p = 0.970).

**Table 2:** Heart rate & HRV before, during and after CPR.

Parameter (mean, SD)	Before resuscitation	During resuscitation	After resuscitation	p-value
**Heart rate**				
Mean [/min]	87 (16)	97 (19)	80 (14)	<0.001
Minimal [/min]	66 (12)	77 (16)	63 (10)	<0.001
Maximal [/min]	116 (18)	116 (22)	108 (18)	<0.001
Reactivity [/min]	50 (14)	39 (14)	50 (14)	<0.001
**Heart rate variability**				
SDNN [ms], overall HRV	117 (91)	92 (56)	93 (37)	<0.001
RMSSD [ms], short-term HRV	58 (48)	58 (62)	59 (42)	0.970

CPR: Cardiopulmonary resuscitation; HRV: Heart rate variability; SDNN: Standard deviation of all normal to normal intervals; RMSSD: Root-mean-square of successive differences; ms: Milliseconds; Before resuscitation: 10min before cardiac arrest; During resuscitation: Duration of cardiac arrest; After resuscitation: 30min after cardiac arrest; Tachycardia: HR >100bpm.

### Dynamic ST-segment and T-wave ECG alterations

**Fig 1:**
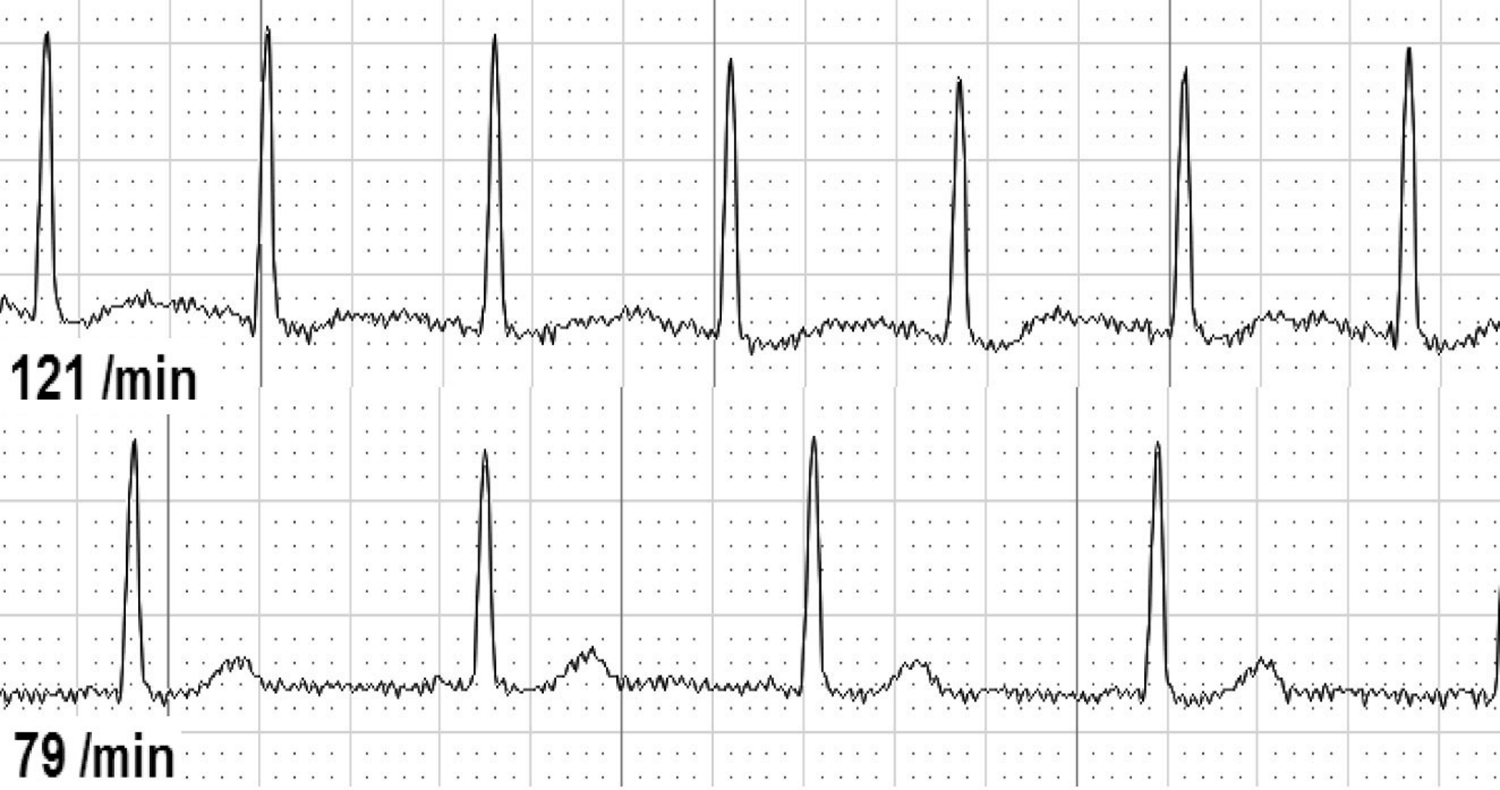
Flattened T-wave during resuscitation (above) vs. normal T-wave.

**Fig 2:**
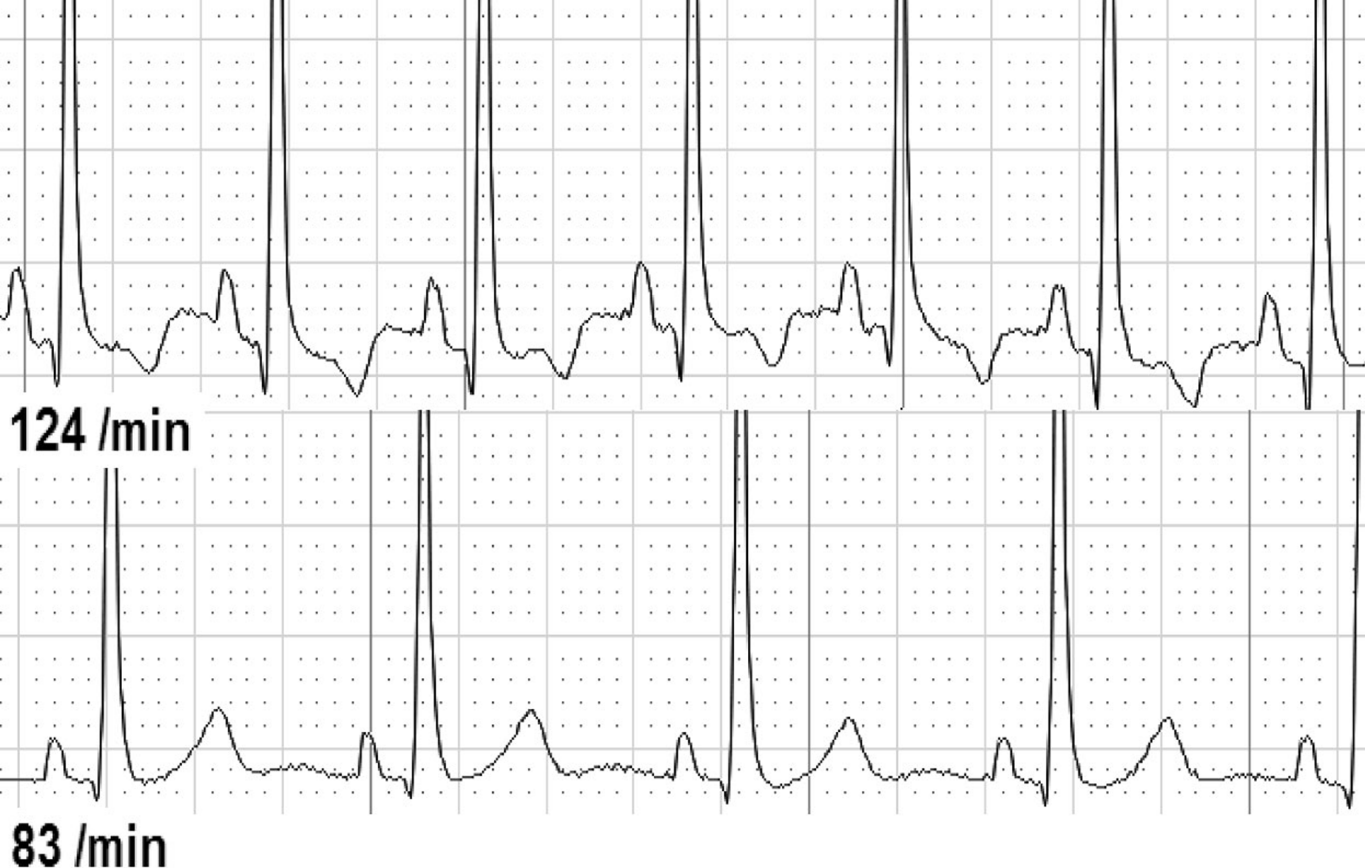
T-inversion before resuscitation (above) vs. normal T-wave.

**Fig 3:**
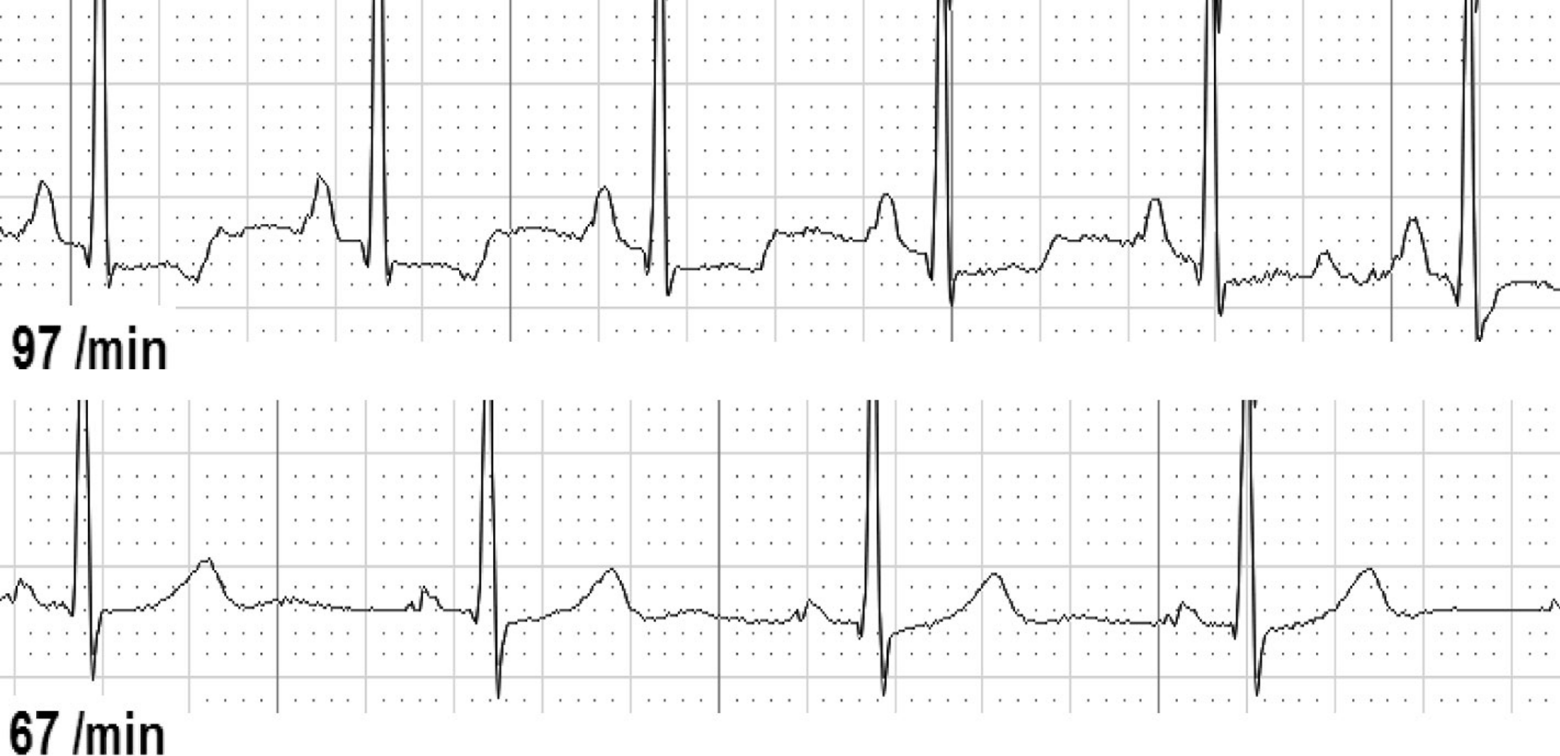
Biphasic T-wave & ST-depression before resuscitation (above) vs. normal T-wave & ST-segment.

**Fig 4:**
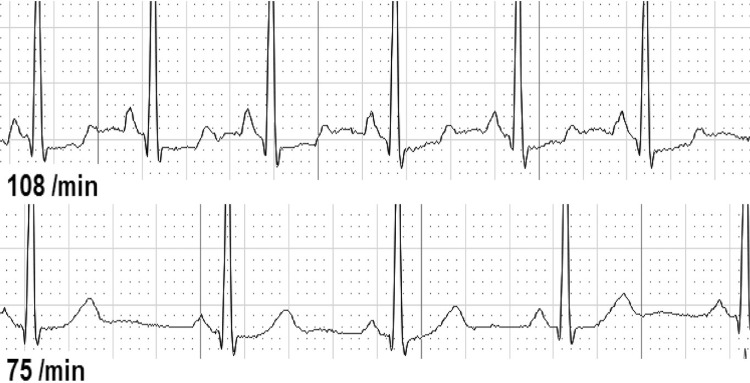
ST-depression during resuscitation (above) vs. normal ST-segment.

**Fig 5:**
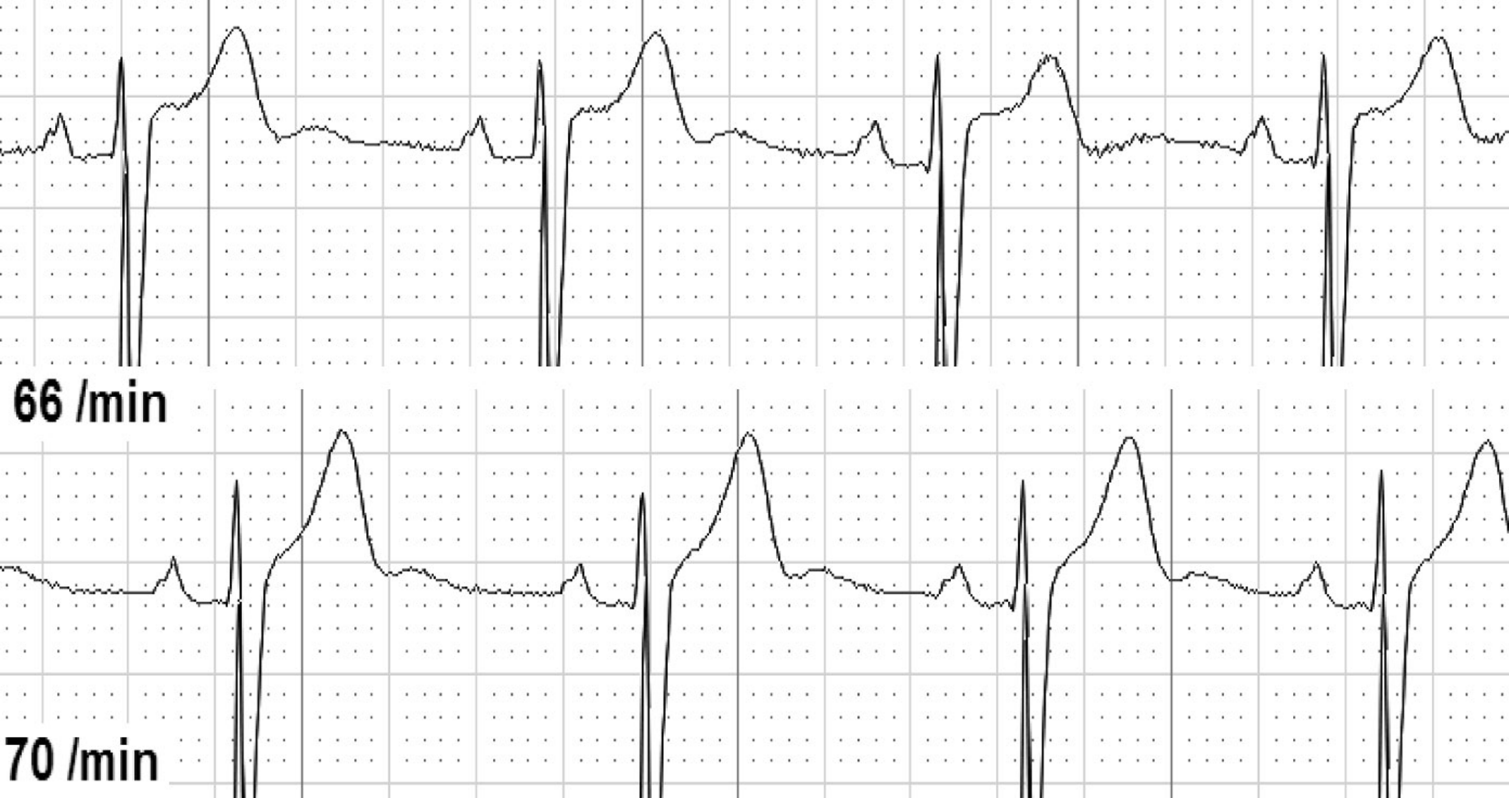
ST-elevation before resuscitation (above) vs. normal ST-segment Numbers/min denote heart rate per minute.

Second, we analyzed ECG changes over the whole-time period from before until after CPR (Table 3). We found either dynamic T-wave or ST-segment abnormalities in 37 of 126 individuals (29.4%). T-waves were temporary flattened in 30 individuals (25.4%, Fig 1), inverted in 7 individuals (5.9%, Fig 2), and biphasic in 2 individuals (1.7%, Fig 3). ST-depressions were found in 8 individuals (6.8%, Fig 4) and one male participant (0.8%, Fig 5) presented ST-elevations during the recording period.

**Table 3:** Overall and subgroup analysis of ECG parameters.

Parameter (mean, SD)	overall	men	women	p-value	no ECG alterations	ECG alterations	p-value
**Heart rate**							
Mean [/min]	81 (12)	78 (12)	82 (12)	0.072	80 (13)	82 (10)	0.250
Minimal [/min]	58 (9)	55 (9)	61 (8)	<0.001	58 (10)	59 (8)	0.700
Maximal [/min]	131 (20)	126 (20)	136 (19)	0.008	128 (20)	136 (18)	0.023
Reactivity [/min]	73 (16)	71 (14)	75 (17)	0.150	70 (15)	78 (15)	0.008
Tachycardia [sec]	724 (585)	527 (1097)	823 (1665)	0.270	714 (1718)	659 (874)	0.840
**Heart rate variability**							
SDNN [ms], overall HRV	109 (32)	119 (36)	102 (26)	0.004	110 (35)	108 (26)	0.770
RMSSD [ms], short-term HRV	60 (39)	71 (50)	52 (26)	0.009	63 (44)	58 (29)	0.320
**ECG stress parameters**							
ECG alterations, n (%)	37 (29.4%)	12 (21%)	25 (36%)	0.080			
ST-segment depression, n (%)	8 (6.8%)	5 (10%)	3 (5%)	0.280			
ST-segment elevation, n (%)	1 (0.8%)	1 (2%)	0 (0%)	0.260			
Inverted T-wave, n (%)	7 (5.9%)	2 (4%)	5 (8%)	0.390			
Flattened T-wave, n (%)	30 (25.4%)	9 (17%)	21 (32%)	0.072			
Biphasic T-wave, n (%)	2 (1.7%)	1 (2%)	1 (2%)	0.860			

CPR: Cardiopulmonary resuscitation; HRV: Heart rate variability; SDNN: Standard deviation of all normal to normal intervals; RMSSD: Root-mean-square of successive differences; ms: Milliseconds; Before resuscitation: 10min before cardiac arrest; During resuscitation: Duration of cardiac arrest; After resuscitation: 30min after cardiac arrest; Tachycardia: HR >100bpm, ECG: Electrocardiogram

### Differences by gender and by occurrence of ECG alterations

In a next step, we compared these changes between male and female rescuers, and between rescuers with and without ECG alterations (Table 3A). Compared to males, female participants had a significantly higher maximal heart rate (136 bpm vs. 126 bpm, p = 0.008) and significantly lower SDNN (102 vs. 119 ms2, p = 0.004) and RMSSD values (52 vs. 71 ms2, p = 0.009). Also, 36% of females showed abnormalities in either the T-wave or the ST-segment compared to 21% in males (p = 0.080).

Participants showing ECG-alterations had a significantly higher maximal heart rate (136 vs. 128 bpm, p = 0.023) and heart rate reactivity (78 vs. 70 bpm, p = 0.008) as well as lower HRV values (SDNN 108 vs. 110ms, p = 0.770, RMSSD 58 vs. 63ms, p = 0.320).

### Differences by leadership designation and by chest compression

Finally, we investigated differences by leadership designation and by chest compression only focusing on ECG changes during the resuscitation phase (Table 4). We found no significant differences in SDNN, RMSSD and heart rate between participants with and without leadership designation.

**Table 4:** Effect of leadership and chest compressions on ECG parameters during resuscitation.

Parameter (mean, SD)	no leadership designation	leadership designation	p-value	no chest compression	chest compression	p-value
**Heart rate**						
Mean [/min]	95 (18)	88 (22)	0.240	89 (16)	97 (19)	0.020
Minimal [/min]	77 (15)	71 (16)	0.270	73 (13)	77 (16)	0.270
Maximal [/min]	114 (20)	104 (18)	0.130	105 (17)	119 (19)	<0.001
Reactivity [/min]	37 (13)	33 (6)	0.320	32 (10)	42 (12)	<0.001
Tachycardia [sec]	41 (71)	7 (14)	0.150	20 (50)	54 (83)	0.016
**Heart rate variability**						
SDNN [ms], overall HRV	84 (41)	76 (28)	0.520	76 (23)	95 (52)	0.019
RMSSD [ms], short-term HRV	49 (46)	32 (11)	0.230	47 (30)	52 (58)	0.610

CPR: Cardiopulmonary resuscitation; HRV: Heart rate variability; SDNN: Standard deviation of all normal to normal intervals; RMSSD: Root-mean-square of successive differences; ms: Milliseconds; Before resuscitation: 10min before cardiac arrest; During resuscitation: Duration of cardiac arrest; After resuscitation: 30min after cardiac arrest; Tachycardia: HR >100bpm.

Students who performed chest compressions showed significantly higher mean heart rate (97 vs. 89bpm, p = 0.020), heart rate reactivity (42 vs. 32bpm, p<0.001) and SDNN (95 vs. 76ms, p = 0.019) during the resuscitation phase. However, when we analyzed the whole simulation, these differences did not reach statistical significance.

### Association of stress measures and performance

Group-performance, assessed by means of hands-on time, was associated with both heart rate and heart rate variability during resuscitation (Table 5). Maximal heart rate (regression coefficient 0.25, 95%CI 0 to 0.5, p = 0.046), heart rate reactivity (regression coefficient 0.7, 95%CI 0.32 to 1.07, p<0.001) and tachycardia (regression coefficient 7.04, 95%CI 0.01 to 14.07, p = 0<0.050) during resuscitation were significantly associated with hands-on time at 180 seconds after start of CPR. SDNN during resuscitation showed a trend towards positive association with hands-on time, which did however not reach statistical significance (regression coefficient 0.1, 95%CI -0.02 to 0.22, p = 0.094). Performance in the group of the individual presenting ST-elevations was significantly decreased (regression coefficient -63.11, 95%CI -110.90 to -15.32, p = 0.010), reaching only 35 seconds hands-on time after 180 seconds.

**Table 5:** Linear regression analysis of stress measures with hands-on time*.

Parameter	Univariate regression coefficient (95%CI)	p-value	Multivariate regression coefficient (95%CI)**	p-value
**Overall**				
**Heart rate**				
Mean [/min]	-0.02 (-0.41, 0.37)	0.924	-0.03 (-0.44, 0.39)	0.904
Minimal [/min]	-0.18 (-0.71, 0.35)	0.507	-0.22 (-0.79, 0.36)	0.459
Maximal [/min]	-0.07 (-0.3, 0.17)	0.580	-0.08 (-0.33, 0.17)	0.530
Reactivity [/min]	-0.05 (-0.36, 0.25)	0.739	-0.06 (-0.38, 0.26)	0.707
Tachycardia [/100 sec]	0.14 (-0.18, 0.46)	0.384	0.14 (-0.19, 0.46)	0.417
**Heart rate variability**				
SDNN [ms], long-term HRV	0.07 (-0.08, 0.22)	0.365	0.08 (-0.08, 0.23)	0.343
RMSSD [ms], short-term HRV	0.01 (-0.1, 0.13)	0.805	0.02 (-0.11, 0.14)	0.775
**ECG stress parameters**				
All	-1.77 (-11.63, 8.09)	0.723	-1.53 (-11.73, 8.67)	0.767
ST-segment depression	1.87 (-16.18, 19.91)	0.838	2.53 (-16.04, 21.1)	0.788
ST-segment elevation	-63.11 (-110.90, -15.32)	0.010	-64.84 (-113.74, -15.93)	0.010
Inverted T-wave	-8.20 (-27.33, 10.90)	0.397	-8.39 (-27.89, 11.12)	0.396
Flattened T-wave	-6.87 (-17.46, 3.73)	0.202	-7.38 (-18.4, 3.64)	0.187
Biphasic T-wave	-14.8 (-49.73, 20.13)	0.403	-14.77 (-50.24, 20.7)	0.411
**During resuscitation**				
**Heart rate**				
Mean [/min]	0.15 (-0.12, 0.42)	0.269	0.14 (-0.14, 0.43)	0.319
Minimal [/min]	-0.05 (-0.37, 0.28)	0.781	-0.07 (-0.4, 0.27)	0.701
Maximal [/min]	0.25 (0, 0.5)	0.046	0.27 (-0.01, 0.54)	0.055
Reactivity [/min]	0.7 (0.32, 1.07)	<0.001	0.82 (0.4, 1.24)	<0.001
Tachycardia [/100 sec]	7.04 (0.01, 14.07)	0.050	7.3 (0, 14.6)	0.050
**Heart rate variability**				
SDNN [ms], long-term HRV	0.1 (-0.02, 0.22)	0.094	0.1 (-0.02, 0.22)	0.101
RMSSD [ms], short-term HRV	0.04 (-0.07, 0.14)	0.474	0.04 (-0.07, 0.15)	0.477

*hands-on time during the first 180 seconds after start of CPR

**adjusted for gender, chest compression and leadership designation

CPR: Cardiopulmonary resuscitation; HRV: Heart rate variability; SDNN: Standard deviation of all normal to normal intervals; RMSSD: Root-mean-square of successive differences; ms: Milliseconds; Before resuscitation: 10min before cardiac arrest; During resuscitation: Duration of cardiac arrest; After resuscitation: 30min after cardiac arrest; Tachycardia: HR >100bpm, ECG: Electrocardiogram

After adjustment for gender, leadership designation and chest compression, the association of maximal heart rate with hands-on time was no longer significant, while the association of heart rate reactivity and tachycardia with hands-on time remained significant.

Further data can be found in S1 Table.

## Discussion

In this study, using a simulated cardiac arrest scenario as a model of acute stress, we investigated the effect of stress on ECG-findings of rescuers and on different measures of sympathetic activation during cardiopulmonary resuscitation and their association with CPR performance. Our aim was to examine whether stress leads to ECG alterations and is associated with gender or team performance. We found that almost a third of all participants showed dynamic ECG alterations, either in the ST-segment (depression or elevation) or the T-wave (flattened, inverted or biphasic). These changes were transient and occurred at different time points before, during and after resuscitation.

Acute mental stress, through effects of cortisol and adrenaline, leads to temporary vascular endothelial dysfunction which can result in ischemia [12]. It is well researched that stress is a common risk factor for cardiac events in patients with underlying coronary heart disease (CHD) [13]. However, to our knowledge, this is the first study, which shows that stress, induced by the performance of CPR, leads to dynamic ECG changes in healthy medical students. Simulator-based resuscitation scenarios may provoke mental stress and influence cardiac physiology more than previously believed as evidenced by the ECG alterations found in our study. Dynamic ECG-changes can sometimes be seen non-specifically in healthy young adults during physical activity [14, 15]. In our study, we found that individuals who showed dynamic ECG alterations also had a lower HRV and significantly higher HR. This suggests that these rescuers suffered not only from autonomic cardiac dysregulation (low HRV) but also showed signs of acute myocardial stress. Hence, we consider the ECG dynamics of our participants as likely stress-induced.

Early research shows that cardiovascular reactivity to mental stress differs significantly between gender with females showing a stronger heart rate response than males due to decreased vagal activity [16]. In this study, females did indeed show an overall stronger reaction than males in regard to ECG changes. Especially ST-segment and T-wave alterations were mainly observed in female subjects. These results are in line with previous studies showing that female rescuers perceive higher stress and negative emotions during resuscitation than males [5]. From the field of behavior research, there is evidence that the physiological reaction to stress is potentially connected with self-esteem levels [17]. Further research should investigate if self-esteem can partly explain the gender differences found in our study.

In terms of CPR performance of inexperienced rescuers, females have shown less leadership behavior, less hands-on time and are generally less proactive than males [18, 19]. For this reason, we additionally searched for a possible association of individual stress parameters with group performance. In past studies, elevated levels of perceived stress were often associated with decreased CPR performance and higher heart rate with increased hands-on time [5, 9, 20]. In line with previous studies, tachycardia was associated with better group performance (hands-on time) in our analysis. As tachycardia remained significantly associated with hands-on time when adjusted for chest compressions, a higher heart rate may still reflect higher physical activity or other factors, such as higher motivation. Thus, this might indicate that HR is not a reliable stress marker during resuscitation due to confounding. The impact of motivation and other emotions on resuscitation performance needs to be examined in further research.

Higher SDNN values, as a possible surrogate for low mental stress, showed a trend towards a positive correlation with CPR performance. This may indicate that mental stress may have impaired optimal performance in this study. Indeed, it is well known from other work areas that higher loads of mental stress may interfere with workflow and compromise performance [21]. The United States Air Force for example, conducted several studies in this research area with battlefield airmen that are highly dependent on functioning in stressful and potentially life-threatening environments. With the aid of developed stress inoculation trainings, these soldiers are able to train stressful events and adapt to these scenarios. Transferred into a medical context, a recurrent training of resuscitation scenarios might lead to a decreased stress reaction, and hereby be able to improve CPR performance in highly stressed rescuers.

However, it has also been shown that stress can improve task performance, depending on individual perception of the task to perform and coping strategies[22]. Research shows that stress may help mobilize energy and enhance the focus on a task, thus “protecting” performance [23]. In addition, emotional stressors during CPR training increase long-term retention of applied knowledge, indicating that there is probably an amount of stress that is needed for learning [24]. Most probably, the effect of mental stress on CPR performance is highly individual and under the influence of other human factors such as personality, a relationship that needs to be examined in future research.

The primary strength of our study is the use of different objective stress measures across different time-points during a standardized CPR situation. The simulator setting created identical conditions for all participants and the study was based on a cohort of medical students with the same professional experience, preventing potential biases by different levels of knowledge. Some limitations of this study need consideration. Technical aspects of ECG recording, such as positioning of the electrodes and upper-body movement during the simulation may have influenced our results. ST- and T-wave abnormalities were assessed using a 3-lead ECG device, which might be insufficient for accurate diagnostic in comparison to a standard 12-lead device used in clinical practice. Additionally, given the fact that our study was performed in a simulation setting, the participants might have perceived the resuscitation setting less stressful than real-life scenarios. However, in previous studies, resuscitation scenarios in high fidelity simulators were experienced as realistic and highly stressful [25, 26]. Finally, our study was performed only on medical students, limiting our results, which cannot be generalized to more experienced medical staff, especially emergency and intensive care physicians.

## Conclusions

CPR causes significant ECG alterations in healthy medical students with ST-segment and T-wave abnormalities, with more pronounced effects in females. Clinical implications of these findings need to be further investigated.

## Supporting information

S1 Table Anova. Further data for Anova analysis.
(XLS)
